# New Frontiers in Prostate Cancer Treatment: Are We Ready for Drug Combinations with Novel Agents?

**DOI:** 10.3390/cells9061522

**Published:** 2020-06-22

**Authors:** Gaetano Aurilio, Alessia Cimadamore, Matteo Santoni, Franco Nolè, Marina Scarpelli, Francesco Massari, Antonio Lopez-Beltran, Liang Cheng, Rodolfo Montironi

**Affiliations:** 1 Medical Oncology Division of Urogenital and Head and Neck Tumours, IEO, European Institute of Oncology IRCCS, 20141 Milan, Italy; franco.nole@ieo.it; 2 Section of Pathological Anatomy, Polytechnic University of the Marche Region, School of Medicine, United Hospitals, 60126 Ancona, Italy; alessiacimadamore@gmail.com (A.C.); m.scarpelli@univpm.it (M.S.); r.montironi@univpm.it (R.M.); 3 Oncology Unit, Macerata Hospital, 62012 Macerata, Italy; mattymo@alice.it; 4 Division of Oncology, S. Orsola-Malpighi Hospital, 40138 Bologna, Italy; francesco.massari@aosp.bo.it; 5 Department of Surgery, Cordoba University Medical School, 14071 Cordoba, Spain; em1lobea@gmail.com; 6 Department of Pathology and Laboratory Medicine, Indiana University School of Medicine, Indianapolis, IN 46202, USA; liang_cheng@yahoo.com

**Keywords:** prostate cancer, metastatic castration-resistant prostate cancer, DNA damage repair, ARS inhibitors, PARP inhibitors, immune checkpoint inhibitors, PSMA-inhibition, drug combinations

## Abstract

Medical treatment for metastatic castration-resistant prostate cancer (mCRPC) patients has progressively been evolving from a nonspecific clinical approach to genomics-oriented therapies. The scientific community is in fact increasingly focusing on developing DNA damage repair (DDR) defect-driven novel molecules, both as single-agent therapy and in combined treatment strategies. Accordingly, research is under way into combined drug therapies targeting different pathways, e.g. androgen receptor signaling (ARS) and poly (adenosine diphosphate [ADP]-ribose) polymerase (PARP) enzymes, immune checkpoint (IC) and PARP, IC, and ARS, and prostate-specific membrane antigen (PSMA). In an attempt to formulate evolving treatment paradigms in mCRPC patients, here we selected clinical research into patients undergoing therapies with emerging molecules, with particular emphasis towards PARP-, IC-, and PSMA-inhibitors. In order to focus on those molecules and drug combinations most likely to be translated into routine clinical care in the near future, we selected only those clinical studies currently recruiting patients. A PubMed search focusing on the keywords “prostate cancer”, “metastatic castration-resistant prostate cancer”, “DDR pathways”, “ARS inhibitors”, “PARP inhibitors”, “IC inhibitors”, “PSMA-targeting agents”, and “drug combinations” was performed.

## 1. Background

The androgen receptor (AR) pathway is the key driver for PCa growth and subsequent progression. Two potent AR-signaling (ARS) inhibitors, abiraterone acetate and enzalutamide, were first developed in patients with metastatic castration-resistant PCa (mCRPC) [1,2], and along with the taxanes docetaxel and cabazitaxel and the alpha emitter radium-223 dichloride represent current best practice in this disease in many Western countries. This body of drugs has certainly improved the oncological outcomes of PCa patients, although disease progression eventually occurs during treatments and the treatment choice beyond progression after 2–3 previous therapy lines is often challenging.

Next-generation sequencing (NGS) data of a large PCa patient population have led to the detection of germline mutations in DNA damage repair (DDR) genes equal to 11.8% and 4.6% in metastatic and localized disease patients, respectively [3]. Somatic mutations in DDR genes occur instead in 20–25% of mCRPC patients [4]. Germline DDR gene mutations occurring most frequently in *BRCA2* gene have been associated with more aggressive disease features, a higher risk of relapse, and overall a poor prognosis, independently of prostate-specific antigen (PSA) value and/or cellular differentiation score [3]. Along these lines, DDR genes such as *ATM*, *CHEK2*, *BRCA1*, and *BRCA2* are largely investigated as putative biomarkers in PCa patients. Phase 1-2 studies have shown anticancer activity in mCRPC patients harboring DDR gene aberrations (mainly in *BRCA1/2*) and treated with a poly (adenosine diphosphate [ADP]-ribose) polymerase (PARP) inhibitor [5,6]. Further, PARP family members may regulate AR association with chromatin, promote AR function, and sensitize PCa cells to androgen depletion [7]. Some data underline that PARP-1 expression increases during PCa progression as well as under antiandrogen inhibition [8]. In the light of these suggested mechanisms, the potential interplay across PARP-inhibition and ARS-inhibition is currently under investigation.

Specific DDR alterations may favor a high mutational burden, and trigger the expression of specific neoantigens as well as the increase of tumor-infiltrating lymphocytes. This could support the role of immunotherapeutics, especially using agents targeting the programmed cell death protein (PD)-1 receptor or its ligand (PD-L1) [9].

Another line of research on prostate-specific membrane antigen (PSMA)-targeted therapies is opening the way to additional promising treatment strategies. Therefore, research is under way into combined drug therapies targeting different pathways, e.g. ARS and PARP, immune checkpoint (IC) and PARP, IC and ARS, and PSMA.

Considering all this, for our review, we selected clinical research into mCRPC patients undergoing therapies with emerging molecules, with particular emphasis towards PARP-, IC-, and PSMA-inhibitors, in an attempt to formulate evolving treatment paradigms in this disease phase.

## 2. Article Selection

During the planning stage of this review, we were well aware of the parallel research lines in non-metastatic castration-resistant as well as metastatic castration-naïve PCa patients. We decided to focus exclusively on castration-resistant metastatic patients because of the large amount of available data that would permit us to specifically address our research question.

We performed a PubMed search focusing on the keywords “prostate cancer”, “metastatic castration-resistant prostate cancer”, “DNA damage repair pathways”, “ARS inhibitors”, “PARP inhibitors”, “immune checkpoint inhibitors”, “PSMA-targeting agents”, and “drug combinations”.

## 3. DNA Damage Repair Pathways

It is known that human DNA can be impaired via the action of endogenous and exogenous factors. The former include mechanisms of DNA replication, cell division, while the latter refer to ionizing radiation (IR), ultraviolet rays, and chemical agents. These factors can induce DNA damage as single- (SSB) or double-stranded breaks (DSB). The persistence of DNA damage can evolve towards induction of mutations and global alterations of genome sequencing, a milestone in tumor development.

Three mechanisms are involved in DDR. These are: homologous recombination (HR) and non-homologous end joining (NHEJ) pathways for DNA DSB, and the mismatch repair (MMR) pathway—mainly—for SSB [10]. HR acts through S/G2 cell cycle phases and is the more accurate among such pathways, notoriously being error-free. On a practical level, HR is the replacement of a fragment of parental DNA with a segment characterized by a highly similar (i.e., homologous) sequence from a partner DNA. In detail, firstly, the MRN complex (Mre11-Rad50-Nbs1) identifies broken ends cooperating with two kinases. These kinases are ataxia telangiectasia mutated (ATM), which stimulates download signaling by means of the action of checkpoint kinase 2 protein (Chk2), and ataxia telangiectasia Rad3-related protein (ATR). Following this, BRCA1 mediates end resection to predispose the assembly of the RAD51 complex along with BRCA1, BRCA2, and PALB2 in an attempt to commence homology search and strand invasion. Lastly, making use of a sister chromatid, the entire process culminates in the synthesis and ligation of a novel DNA strand [11].

Unlike HR, the NHEJ pathway acts most importantly during G1 phase; it does not use a DNA template and accordingly is thought of as an error-prone mechanism, thereby being l less accurate overall. To proceed with the repair, NHEJ uses short homologous DNA fragments called microhomologies, that are mostly placed on the ends of DSB, and the accuracy of the repair is linked to their degree of compatibility. The heterodimer protein Ku70/80, encoded by the 2 genes *XRCC5* and *XRCC6* genes, recognizes and binds to DSB ends forming a DNA complex mediated by an enzyme known as DNA-PKcs (DNA-dependent protein kinase catalytic subunit). Following this, DNA-end processing implicates removal of damaged nucleotide segments by nucleases, the ARTEMIS protein and p53-binding protein1, and then resynthesis by DNA polymerases. The final step of repair, ligation, is mediated by the DNA ligase IV complex, formed by the catalytic subunit DNA ligase-4 and its cofactor XRCC4 [12]. Insufficient NHEJ can generate telomere fusion and translocations, hallmarks of tumor cells.

The MMR pathway is in turn assigned the role of maintaining genomic stability, repairing SSB of DNA base substitution mismatches and insertion–deletion mismatches generated as a consequence of DNA replication defects that sidestep the proofreading role of DNA polymerases. Genes of the MSH and MLH family in particular cooperate for this DNA repair mechanism. In the early phase, the mismatch identification occurs via the *MSH2* and *MSH6* genes, then the endonuclease PMS2 produces notches near the mismatch, allowing EXO1 to take out the DNA fragment harboring the mismatch. Dysregulation of this pathway may induce the occurrence of point mutations [13].

## 4. Single-Agent Therapy and DDR Pathways Correlation

### 4.1. ARS-Inhibition

Gene alterations occurring in the HR DDR pathway have been investigated in mCRPC patients treated with ARS inhibitors, abiraterone acetate and enzalutamide (Table 1). 

Annala and colleagues conducted a retrospective study in mCRPC patients, mostly receiving ARS inhibitors, to assess the prevalence of germline mutations in 73 PCa genes, among which 22 HR DDR members, and to define correlations with clinical outcomes. The authors showed that 24 (7.5%) of all the patients had deleterious germline mutations—*BRCA2* being the most affected gene—that correlated with poor median time to PSA progression (3.3 months) [14]. In another study, 202 mCRPC patients underwent whole-exome sequencing of 72 genes from circulating tumor DNA, followed by abiraterone acetate or enzalutamide as front-line therapy. The authors observed that patients harboring *BRCA2*/*ATM* deleterious alterations as truncating mutations had significantly shorter time to progression [15]. A retrospective observational study examined the germline DDR gene mutation (gDDRgm) status of 390 metastatic PCa patients, 69% of whom received first-line treatment with abiraterone/enzalutamide. The findings between patients with and without gDDRgm on first-line ARS inhibitors did not show any significant difference in terms of median progression-free survival (PFS) and response rate [16]. Along these lines, a panel of 50 DDRg was retrospectively investigated for germline mutations from 172 mCRPC patients receiving front-line therapy with ARS inhibitors. The results demonstrated that survival outcomes were better in patients with germline *BRCA*/*ATM* mutations than in non-*BRCA*/*ATM* mutated patients [17]. A prospective multicenter study evaluated the role of germline DDR gene mutations (*ATM/BRCA1/BRCA2/PALB2*) in 419 unselected mCRPC patients in an attempt to verify cause-specific survival (CSS) correlations. No significant difference in CSS was observed between carrier patients and non-carriers, except for BRCA2 carriers who showed a significant improvement in CSS. This outcome was maintained when comparing first-line treatment with abiraterone/enzalutamide versus taxanes [18].

### 4.2. PARP-Inhibition

Poly (adenosine diphosphate [ADP]-ribose) polymerase (PARP) enzymes play an important role as regulators of the cell cycle and express their action at the DNA SSB level. When deleterious mutations of *BRCA1* and *BRCA2* occur, the cells in turn induce a PARP enzyme stimulation to allow the maintenance of the cell cycle. Among the PARP family members, only PARP1 and PARP2 enzymes are implicated in the DDR mechanism, thus they are the target of anti-PARP drugs. The use of PARP inhibitors (PARPi) directly affects the mechanisms of SSB repair that accordingly evolves in DSB, finally leading to cancer cell death. This phenomenon is known as synthetic lethality. In other words, two hits that are usually not fatal when occurring alone become lethal for the cancer cells when acting together.

Here reported are the completed trials with PARPi, as follows (Table 1).

A phase I trial was conducted in patients with solid malignancies receiving olaparib, initially in tumors that were not *BRCA1/2* mutated; subsequently in the expansion cohort, *BRCA1/2* mutations were mandatory. Sixty patients were treated, 22 of whom had a *BRCA1/2* mutation. The findings indicated that only mutated patients on olaparib benefited, and it was noted one *BRCA2* carrier with mCRPC experienced lasting PSA response and resolution of bone metastases [5]. Building on these data, in a phase II trial (TOPARP-A), olaparib 400 mg twice a day was administered to 50 heavily pretreated mCRPC patients. A total of 16 patients had responses (33%), and in 14 of these patients, DDR gene dysregulations (mostly *BRCA2* and *ATM*) were detected. The authors underlined the high percentage of response in this specific disease setting [19]. Another recently-published phase II study (TRITON2) used rucaparib 600 mg orally twice a day in mCRPC patients harboring non-*BRCA* DDR gene alterations. Patients were previously treated with one or two lines of ARS inhibitor and one line with a taxane-based chemotherapy schedule. A low percentage (<10%) of radiographic/PSA response was reported in the majority of patients who expressed alterations in *ATM*, *CDK12*, and *CHEK2* genes, while patients with other gene affections, e.g., *PALB2* and *RAD51B*, had sometimes better and lasting responses [20]. The publication of the phase 2 multicenter open-label TOPARP-B study also appeared recently. Seven hundred patients with mCRPC progressing to 1–2 lines of taxane-based chemotherapy schedule were screened for DDR gene aberrations. From 161 mutated patients, 98 were randomized (1:1) and then treated with olaparib at a dose of 400 or 300 mg twice daily. Composite response, defined as any radiological and/or biochemical (PSA/CTC (circulating tumor cells) response, was observed in 54% and 39% of the patients with olaparib 400 and 300 mg, respectively. Grade 3–4 anemia occurred in approximately one third of patients in each group [6]. It should be mentioned that Marshall and coworkers retrospectively studied 23 consecutive mCRPC patients with pathogenic mutations (*BRCA1/2* in 17 patients and *ATM* in 6) in order to understand whether the type of mutated gene may affect the olaparib response. The authors noted that patients *BRCA1/2* mutated had longer mPFS than those with *ATM* mutated (12.3 vs. 2.4 months, *p* = 0.004) [21].

Very recently, the phase III PROfound trial testing olaparib versus enzalutamide/abiraterone acetate was published. The study involved the inclusion of mCRPC patients harboring a qualifying tumor gene mutation in the HR DDR pathway (*BRCA1/2* or *ATM* (cohort A), one of 12 other genes (cohort B)) and progressed on prior ARSi, previous taxane chemotherapy use was allowed. The primary endpoint was radiographic-PFS (r-PFS) in cohort A. Three hundred and eighty-seven patients were randomized (2:1) to olaparib or enzalutamide/abiraterone acetate (depending on which ARSi was given as prior treatment). In cohort A, the findings showed that patients had a longer median r-PFS under olaparib compared with the control group (7.4 vs. 3.6 months, *p* < 0.001); ORR, median time to pain progression and interim OS also significantly benefitted with the study drug. In cohorts A and B, median r-PFS was 5.8 vs. 3.5 months, respectively (*p* < 0.001). Anemia and asthenia grade >3 were the most frequent adverse events with olaparib [22].

### 4.3. ICI-Inhibition

Genomics data have been used to detect mutations in MMR genes in approximately 3–5% of advanced PCa patients. Some evidence supports the concept that defects in the MMR (MMR-deficient) pathway may lead to a certain genomic instability. This in turn causes a nucleotide mutation that affects the amino acid sequence of a protein, mutation known as nonsynonymous. This mutation can eventually induce the expression of neoantigens responsible for increasing the immunogenicity of cancer [9,25]. MMR damage may generate genetic hypermutability, known as microsatellite instability (MSI). Accumulation of errors during DNA replication that are not corrected by abnormal MMR function triggers the formation of novel microsatellite fragments, that is, repeated sequences of DNA.

The clinical outcome of patients with MSI/MMR deficiency in treatment with PD-1/PD-L1 inhibitors has been investigated (Table 1). In 86 patients with MMR deficiency tumors prospectively enrolled across 12 different cancer types, among which also PCa, objective and durable responses in half of the sample were demonstrated with the anti-PD-1 inhibitor pembrolizumab. Complete response (CR) was observed in 21% of the patients [9]. At the Memorial Sloan Kettering Cancer Center, 32 of 1033 (3.1%) PCa patients who underwent a molecular tumor profiling, harbored MSI/MMR deficiency, 11 of whom received therapy with an immune checkpoint inhibitor (ICI). Six of these ICI patients had a PSA response, 4 of whom along with radiological response, and of note 5 responders had a lasting therapy [23]. Based on these results, the US Food and Drug Administration (FDA) granted the approval of pembrolizumab for all solid tumors, including mCRPC with MMR deficiency or high MSI expression, the first example of agnostic FDA approval.

### 4.4. PSMA-Inhibition

PSMA is a 100 kD membrane-bound glycoprotein overexpressed in both primary and advanced PCa, and widely explored as diagnostic and therapeutic target.

Across PSMA-targeted radioligands under investigation in mCRPC patients, many studies have assessed the beta-emitter lutetium-177 (177Lu-PSMA-617), a radioligand sometimes used on a compassionate-use basis after failure of usual therapies (Table 1). A meta-analysis of 17 studies, including a total of 671 mCRPC patients, was conducted to shed light on efficacy and safety of 177Lu-PSMA-617. The results showed that 46% of patients had PSA reduction >50% (*p* = 0001); from eight studies that allowed the investigators to assess the disease response, partial response (PR) and stable disease (SD) resulted respectively in 37% and 38% of patients; the toxicity profile was negligible [26]. Other radionuclides are currently under intense investigation both as single-agents and as combined therapies.

Data from a phase 2 single-arm study encompassing 119 mCRPC patients treated with PSMA antibody-drug conjugate (a fully human monoclonal antibody) have recently been published. All patients had received abiraterone acetate and/or enzalutamide and the inclusion criteria allowed one prior chemotherapy-line with taxane. In the chemotherapy patient-group, (84 patients) 61% of patients had SD as best response, while in the chemotherapy-naïve group, (35 patients) 69% of patients had SD and 6% had PR; 7-month OS was equal to 92% for both the two groups. Neutropenia and neuropathy grade-3 occurred in 58% of patients, while treatment was discontinued in 62% of patients, mostly due to progressive disease [24].

Chimeric antigen receptor (CAR)-T cell therapy is another novel approach in cancer patients used as monotherapy or in combined therapies. This strategy is based on engineering T cells that produce CARs in an attempt to directly target cancer antigens, overcoming the usual interaction between the major histocompatibility complex and T-cell receptors. In such a way, CAR-T cells would be an innovative and likely more effective cancer therapy. Currently, phase I trials are studying CAR-T cells based-therapy, as discussed below.

## 5. Combined Drug Treatments

### 5.1. Published Clinical Studies

Here are reported some published clinical studies of mCRPC patients treated with different strategies of drug combinations (Table 2).

- *PARPi plus chemotherapy*: A phase 1 dose-escalation study of veliparib combined with paclitaxel and carboplatin was conducted in patients with advanced solid malignancies not responding to previous therapy. Seventy-three patients with documented or probable BRCA mutation were enrolled. The findings demonstrated that the pharmacokinetics of the two chemotherapy drugs was not affected by concomitant veliparib administration. Twenty-two PR and five CR were observed. The toxicity reported was as expected for each drug involved, displaying a good tolerance overall [27].
- *PARPi plus ARSi:* A phase 2 trial with one-to-one randomization was carried out in 148 mCRPC patients—previously stratified by ETS status (a fusion gene that would enhance PARP-1 inhibition)—to evaluate whether the combination between PARP-1 inhibitor veliparib and ARS inhibitor abiraterone acetate (arm B) is superior to ARS blockade alone (arm A). The findings showed that in patients without DDR pathway alterations (wild-type tumors), there were no significant differences between the two arms in terms of PSA response, tumor response, and PFS, and ETS status did not affect the results. Conversely, 80 patients (33 and 47 for arms A and B, respectively) who harbored DDR gene alterations—detected by NGS of tumor samples—had significantly better results in all the objectives assessed than the counterpart wild-type tumors [28].
- *ICI plus PARPi*: In a small phase 2 cohort-study, 17 mCRPC patients who were previously treated with ARS inhibitors and not prescreened for gene alterations of DDR pathways received a combination of durvalumab plus olaparib. Nine of 17 patients experienced responses, all of whom showed a response of PSA reduction >50% and four as tumor reduction. A large part of the patient responders had mutations in DDR genes. Anemia and nausea grade 3–4 occurred in 24% and 12% of patients, respectively [29].
- *PSMAi plus IL-2:* In a phase I dose-escalation study, five mCRPC patients previously receiving chemotherapy conditioning were treated with PSMA-targeted CAR-T cells and continuous infusion of low-dose interleukin 2. The findings successfully showed that two patients engrafted exhibited tumor response as PR along with 50%/70% of PSA reduction; another patient had a minor response. Clinical responses significantly correlated with plasma IL-2 value, and anti-PSMA toxicities did not occur [30].

### 5.2. Ongoing Trials

In recent years, many clinical trials have been designed for interrogating certain multidrug-based therapeutic strategies. Therefore, there are now many ongoing investigations. However, several of these are not recruiting patients. Accordingly, in an attempt to focus on those molecules and drug combinations most likely to be translated into routine clinical care in the next future, we selected only those clinical studies currently recruiting patients (Table 3).

**Figure 1:**
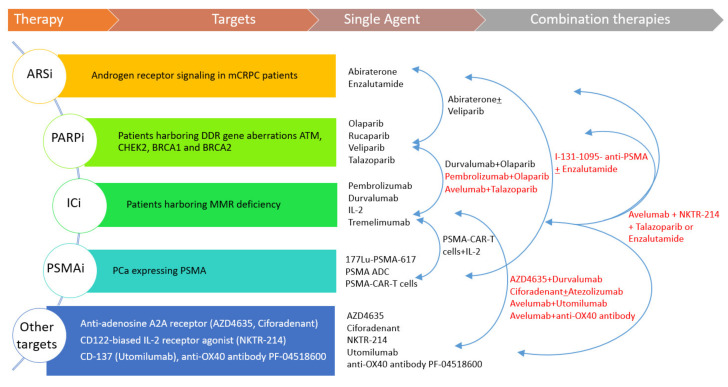
The figure shows the principal classes of therapies investigated in mCRPC patients, their molecular targets, and the single agents and combination therapies discussed (ongoing trials in red).

- ICI- and PARP-inhibition: An anti-PD-1 inhibitor pembrolizumab and PARPi olaparib combination is being investigated in a phase 1b/2 umbrella study (NCT02861573). Preliminary data in 41 molecularly unselected patients previously treated with docetaxel and one ARS inhibitor have shown a PSA response in 13% of the patients. Grade 3–5 treatment-related adverse events occurred in half of the patients, and the authors stated that the safety profile was in line with the spectrum of toxicity of each drug explored. A phase 2 single-arm open-label study (NCT03565991) is evaluating the combination of anti-PD-L1 avelumab plus PARPi talazoparib in patients with locally advanced or metastatic solid tumors—encompassing also patients with mCRPC—with *BRCA1/2* genes or *ATM* gene defects. Talazoparib is a PARPi with the greatest PARP trapping potency (equal to 1, range 1–5) and the longest half-life (90 h) among the PARPi under development. Two hundred patients are expected to be enrolled into the study.
- ICI and anti-adenosine A2A receptor: It is known that adenosine inhibits the anti-cancer function of T-cells and other immune system cells. According to this, some molecules that block adenosine, hampering the binding to its A2A receptor (also known as ADORA2A) and consequently stimulating the immune system response, are under consideration. It follows that the rationale for combining anti-adenosine agents with ICI is to boost the human immune system. A phase 1 open-label dose escalation trial (NCT02740985) is currently investigating oral dosing of AZD4635 (a selective small agent adenosine A2A receptor antagonist) both as a single agent and in combination with anti-PD-L1 durvalumab or other agents in patients with advanced solid tumors, including those with mCRPC. The estimated enrollment is of 307 patients. Ciforadenant is another oral small molecule anti-adenosine A2A receptor on T-cells and other immune cells. In a phase 1/1b open-label study (NCT02655822), ciforadenant is under development as a monotherapy and plus PD-L1 inhibitor atezolizumab in patients with mCRPC or metastatic renal cell carcinoma. The updated estimated enrollment is 336 patients.
- ICI-based combination: The interleukin-2 receptor (IL-2R) is a protein with three different chains (alpha, beta, and gamma), located on the cell surface of certain immune system cells, e.g., lymphocytes, that interacts with the IL-2 cytokine. CD122 is the beta subunit of the IL-2R and is said to play a role in the T cell-mediated immune response. A phase 1b/2 trial (NCT04052204) is investigating the combination of anti-PD-L1 avelumab plus bempegaldesleukin (NKTR-214, a CD122-biased IL-2 receptor agonist) with PARPi talazoparib or ARSi enzalutamide. The study design includes three treatment arms: avelumab plus bempegaldesleukin (arm A) for locally recurrent/metastatic head and neck cancers; avelumab plus bempegaldesleukin plus talazoparib (arm B); and avelumab plus bempegaldesleukin plus enzalutamide (arm C) for mCRPC patients. In combination B, it is planned to enroll subjects with DDR deficiency for phase 2. The updated estimated enrollment is 127 participants. Immunotherapy with avelumab-based combinations is being explored in an open-label phase I/II study (NCT03217747) in patients with advanced malignancies, including both locally-advanced and metastatic PCa patients. The study design includes six treatment arms, in which avelumab is combined with other monoclonal antibodies (utomilumab and anti-OX40 antibody PF-04518600) and in three arms also with radiotherapy. A sample size of 184 subjects is planned to be reached. The MOVIE study is a phase I/II study (NCT03518606) designed to examine a combination consisting of oral metronomic chemotherapy with vinorelbine and a double immune blockade with durvalumab and an anti-cytotoxic T-lymphocyte antigen-4 (CTLA-4) known as tremelimumab in locally advanced/metastatic solid tumors, among which is PCa. It is estimated that 150 participants will be enrolled.
- PSMA-targeting combinations: In a phase I dose-escalation study (NCT04053062), the safety and efficacy of PSMA-specific CAR modified autologous T cells (PSMA-CART cells) is being evaluated in mCRPC patients. Two cohorts of participants have been preplanned, in cohort 1, subjects receive an intravenous single dose of PSMA-CART cells on day 0 after a conditioning regimen (on days -6 to -4) of chemotherapy with cyclophosphamide and fludarabine. Twelve subjects are requested for this study. Another single arm phase I study (NCT03089203) is evaluating the safety and feasibility of dual PSMA-specific/TGFβ-resistant CAR modified autologous T cells (CART-PSMA-TGFβRDN cells) without (cohort 1 and cohort 2) and with (cohort 3) intravenous cyclophosphamide in mCRPC patients. Cohorts 1 and 2 allow the maximum tolerated dose of CART-PSMA-TGFβRDN cells to be identified, while the use of cyclophosphamide in the cohort 3 before CAR-T cells makes use as conditioning chemotherapy. Eighteen patients are estimated to be enrolled into the study. A randomized phase 2 open-label study (NCT03939689) is investigating a radioconjugate radiolabeled with iodine I-131-1095, for delivering iodine cytotoxicity selectively for PSMA-expressing PCa cells, combined with (80 patients) or without (40 patients) enzalutamide in patients with mCRPC. Eligible criteria allow the inclusion of chemotherapy-naïve patients progressing under abiraterone (Figure 1).

## 6. Conclusions and Future Horizons

Medical treatment for mCRPC patients has progressively been evolving from a nonspecific clinical approach to genomics-oriented therapies. In addition to standard approved therapies, in the near future, the treatment landscape of mCRPC patients will probably be enlarged by the introduction of further novel therapeutics such as PARPi and anti-PD-1/PD-L1 inhibitors, beyond those already recently recommended. The scientific community is increasingly focusing on developing DDR defect-driven novel molecules, both as single-agent therapy and in combined treatment strategies. As reported here, the selected drug combinations under development often include immune checkpoint blockers. These new drugs require a preliminary gene determination before starting to be administered, since certain gene aberrations have strictly proven to be predictive of efficacy for a subset of men with mCRPC receiving these classes of drugs. In this context, the phase III, biomarker-selected PROfound trial data underline that in HR DDR gene mutated (*BRCA* and non-*BRCA*) mCRPC patients, the PARPi olaparib is able to significantly prolong rPFS, OS and the time to pain progression, and to increase ORR (33% vs. 2%) versus ARSi patients. It is of interest to note that the olaparib benefit was observed both before and after chemotherapy; the activity of olaparib was also proved in patients with non-*BRCA1/2* gene mutations, although further gene-level confirmation studies are necessary. This is the first published phase III trial using a molecular-driven therapy in mCRPC patients. In the light of this, the genomic sequencing of mCRPC patients is beginning to become a reality and accordingly a medical need. In vitro data have shown enzalutamide to affect the expression of some HR genes in CRPC cells, inducing HR lability and thus a state of BRCAness; the subsequent use of olaparib has caused cell death by inhibition of DDR, both in cell cultures and in PCa xenograft mice models [31]. These data may further suggest the possibility to explore treatment sequencing in mCRPC patients, for instance, a sequence of ARSi followed by PARPi [8] and afterwards the other ARSi [7], and certainly strengthen the concept of genomic testing. The genetic assessment should become an independent step within the diagnostic initial workup of a metastatic PCa patient, irrespective of disease diffusion, histological features and healthy status, when considering the possibility during the natural disease course of therapeutic resistance occurrence and of developing aggressive histological variants. The latter, occurring in approximately 30% of PCa patients, might harbor somatic/germline DDR aberrations, and thus potentially targetable.

The therapeutic scenario becomes even more appealing when considering combinations between DNA-damaging cytotoxic agents i.e., platinum agents and PARPi that have shown antitumor activity along with good tolerability and without pharmacokinetic interactions. This synergy is evident in a recent clinical trial in which veliparib potentiated carboplatin-induced DNA impairment increasing the anticancer activity [27].

The encouraging results from phase 1 to 3 clinical studies with PARPi here presented seem to suggest to clinicians the utility to perform a basal screening for detecting at least *BRCA1/2* aberrations. Similarly, the complete tumor remissions on ICI-based therapy in mCRPC patients who have MSI/MMR deficiency suggest that the initial molecular tumor profile for screening DDR defects should also include the aberrations of the MMR pathway.

About that, as for mCRPC patients, NCCN guidelines recommend tumor testing for HR DDRgm and invite to consider tumor testing for MSI or dMMR. Accordingly, as second-line treatment after first-line abiraterone/enzalutamide or docetaxel, olaparib is mentioned as an option in HR gene mutated patients (category 2B) whilst pembrolizumab in the case of MSI-H or dMMR (category 2B), the latter also for subsequent treatment lines (NCCN Guidelines Version 1.2020 Prostate Cancer). In addition, for EAU guidelines, known genetic alterations, known histological variants, and DNA repair deficiency as well are part of the treatment selection, though the position of the expert panel would seem less strong.

As regarding the correlation between DDR gene mutations and clinical outcomes in mCRPC patients receiving ARS inhibitors as single-agent therapy, the data illustrated are rather discordant and indicate non-one-way implications. Further insights are therefore needed.

**Figure 2:**
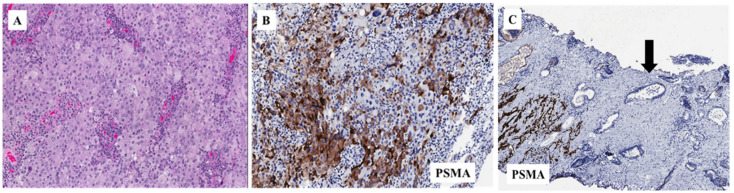
Prostatic adenocarcinoma from a castration-resistant patient (**A**: H&E (20× magnification), positive for PSMA (20× magnification) (**B**). (**C**): non-neoplastic prostatic glands negative for PSMA staining (arrow) (10× magnification).

The studies here presented on PSMA-based therapy in mCRPC patients might suggest in the near future a role for this approach in the advanced disease phase as maintenance therapy after prior tumor response (i.e., taxane-based chemotherapy), there being a high percentage of stable disease obtained with PSMA inhibition. In this regard, 177Lu-PSMA-617 data are promising; however, randomized clinical trials are warranted in order to appropriately define the role of this radioligand therapy. Of interest on an exclusively speculative molecular basis, our group detected high PSMA overexpression on tissue samples of PCa in a castration-resistant patient. Of note, PSMA was not present in the normal prostate tissue [32] (Figure 2).

An attempt with maintenance therapy in a randomized double-blind placebo-controlled phase II trial is being studied with darolutamide, a second-generation oral AR antagonist. Patients with mCRPC receiving first-line with ARSi and non-progressive on a second line taxane-based chemotherapy are eligible for the study. The primary endpoint is rPFS at 12 weeks after treatment initiation, final data collection is estimated for December 2020 [NCT02933801].

In the context of treatment sequencing hypotheses, some researchers have reported the clinical outcome of three heavily pretreated cases of mCRPC patients who received anti-PD-1 inhibitors following bipolar androgen therapy and enzalutamide. All three cases were microsatellite stable, although the authors did not exclude methodological defects. Surprisingly, two patients had a partial and lasting disease response, and one patient achieved an early and complete PSA response [33]. Although of speculative interest, the “priming” role of combined androgen therapy plus enzalutamide before ICI therapy, and the benefit of ICIs in such an immunologically unusual condition (no MMR deficiency) may help in guiding clinicians towards another potential treatment sequence. In this regard, a single-arm phase II study (NCT03554317) is evaluating the effects of androgen ablative therapy with a GnRH analogue administered every 4 weeks for a lead-in period of 12 weeks and then combined with nivolumab every 4 weeks in mCRPC patients who have progressed to one or both ARSi. A previous line of taxane is allowed.

In recent years, emerging interest has also been directed towards the study of fecal microbiota in PCa patients. Preclinical evidence suggests that examining the cellular components of the fecal microbiota may allow us to select which patients undergo anti-PD-1/PD-L1 agents [34]. Therefore, controlled studies of mycobacterial immunotherapy would be of benefit.

To conclude, the evolving treatment paradigms of mCRPC patients will likely be oriented by a genomics-based approach. Performing routinely genomic testing could optimize the integration of novel drugs within the current armamentarium, improving the selection of mCRPC patients and identifying the molecular basis for sequencing novel treatments. Several themes however still remain to be clarified by precision oncology, for instance, the role of tumor heterogeneity, the molecular underpinnings of treatment resistance, and the cross-reactivity of carrier gene mutations, besides controversies on the tissue quality for genetic testing, panels to use, and how to interpret the findings of a molecular tumor board.

## Figures and Tables

**Table 1:** Published clinical studies in metastatic castration-resistant prostate cancer (mCRPC) patients exploring the correlation between DNA damage repair (DDR) pathways and single-agent therapies with androgen receptor-signaling inhibitors (ARSi), poly (adenosine diphosphate [ADP]-ribose) polymerase inhibitor (PARPi), immune checkpoint inhibitors (ICI), and prostate-specific membrane antigen inhibitors (PSMAi).

Drug	Exploratory analysis	N	Design	Phase	Findings [Ref]
ARSi Abi./Enza.	Sequencing of 73 PCa genes	319	R	NA	7.5% of pts with germline mutations (> BRCA2) → 3.3 mo mPSA progression [14]
ARSi Abi./Enza.	WES of 72 driver PCa genes	202	R	NA	BRCA2/ATM defective → shorter TTP [15]
ARSi Abi./Enza.	Search of gDDRgm	390	R	NA	Carriers vs. non-carriers: no difference as PFS and RR [16]
ARSi Abi./Enza.	Search of gDDRm in 50 genes	172	R	NA	BRCA/ATM mutations → better survival [17]
ARSi Abi./Enza.vs Taxanes	Search of gDDRm in 107 genes	419	P	2	BRCA2 mutations → better CSS (outcome maintained comparing ARSi vs. Taxanes) [18]
PARPi Olaparib	Search of BRCA1/2 mutation	60	P	1	22 pts with BRCA1/2 mutation: benefit on Olaparib [5]
PARPi Olaparib (TOPARP-A)	WES; transcriptome analysis	50	P	2	14 out of 16 responders had DDR defects [19]
PARPi Rucaparib (TRITON2)	Mandatory non-BRCA DDR gene defects	78	P	2	ATM, CDK12, CHEK2: <10% response. PALB2, RAD51B: better and lasting response [20]
PARPi Olaparib (TOPARP-B)	Screening for DDR gene defects	700	P	2	98 mutated pts received Olaparib 400/300 mg: 54%/39% tumor response [6]
PARPi Olaparib	Gene defects and response	23	R	NA	BRCA1/2 vs. ATM: 12 vs. 2 mo mPFS [21]
Olaparib vs Abi./Enza. (PROfound)	HR DDRgm: A (BRCA1/2 or ATM), B (other genes)	387	P	3	Cohort A: mr-PFS (pe): 7.4 vs. 3.6 (*p* < 0.001) Cohorts A and B: mr-PFS: 5.8 vs. 3.5 (*p* < 0.001) [22]
Anti-PD-1 Pembrolizumab	MMR deficiency	86	P	2	50% of responders (21 CR) [9]
Anti-PD-1/PD-L1 therapy	Molecular tumor profile	1033	R	NA	3.1% of pts had MSI/MMR deficiency→ 6/11 had responses [23]
PSMA ADC	PSMA expression on CTC, NE markers	119	P	2	Chemo-group: 61% SDChemo-naive group: 69% SD, 6% PR 7-mo OS: 92% for both the two groups [24]

Abbreviations: DDR, DNA damage repair; Ref., references; N, size; D, design; ARSi, androgen receptor signaling inhibitor; Abi., abiraterone acetate; Enza., enzalutamide; PCa, prostate cancer; mCRPC, metastatic castration-resistant prostate cancer; R, retrospective; NA, not applicable; pts, patients; mo, months; WES, whole-exome sequencing; P, prospective; TTP, time to progression; gDDRgm, germline DNA damage repair gene mutation; PFS, progression-free survival; RR, response rate; CSS, cause-specific survival; ORR, objective response rate; CTC, circulating tumor cells; mPFS, median progression-free survival; A, cohort A; B, cohort B; MMR, mismatch repair; ST, solid tumors; CR, complete response; pe, primary endpoint; mr-PFS, median radiographic-progression-free survival; anti-PD-1, anti-programmed cell death protein-1; anti-PD-L1, anti-programmed cell death protein-1 ligand; ICI, immune checkpoint inhibitor; MSI, microsatellite instability; Lu-PSMA, lutetium-prostate-specific membrane antigen; DRC, disease-control rate; ADC, antibody-drug conjugate; SD, stable disease; PR, partial response; OS, overall survival.

**Table 2:** Prospective clinical studies with combined drug therapies in mCRPC patients.

Drugs	Inclusion Criteria	Objectives	N	Phase	Findings [Ref]
PARPi Veliparib + Chemo. (PTX + CBDCA)	advanced solid tumors treated with ≤3 prior regimens, BRCA status not mandated	P. obj.: side effects Recommended phase II dose S. obj.: ORR	73	1	22 PR (1 in mCRPC pt) and 5 CR. Overall good tolerability Chemo. PK was not affected by PARPi [27]
ARSi Abiraterone + PARPi Veliparib	mCRPC, up to two prior chemotherapy regimens	P. obj.: PSA and RR, ETS response prediction; S. obj.: PFS, biomarkers	148	2	Arm A (ARSi) vs. Arm B (combo): no difference in wt pts; pts with DDR defects vs. wt: significantly better outcomes [28]
Anti-PD-L1 Durvalumab + PARPi Olaparib	Prior 1-2 ARSi; no preplanned DDR prescreening	P. obj.: clinical efficacy; S. obj.: ORR, PSA response, DDR status, biomarkers	17	2	9 /17 with PSA response, 4 of whom also disease response (large part of responders had DDRgm) [29]
PSMA-targeted CAR-T cells + IL-2	mCRPC	P. obj.: safety of PSMA-targeting with transduced T cells S. obj.:PSA response	5	1	2 PR with PSA response [30]

Abbreviations: N, size; D, design; PARPi, poly (adenosine diphosphate [ADP]-ribose) polymerase inhibitor; Chemo., chemotherapy; PK, pharmacokinetics; PR, partial response; CR, complete response; ARSi, androgen receptor signaling inhibitor; P. obj., primary objectives; RR, response rate; ETS, a fusion gene; S. obj., secondary objectives; PFS, progression-free survival; mCRPC, metastatic castration-resistant prostate cancer; wt, wild-type tumors; DDR, DNA damage repair; ORR, Objective response rate; anti-PD-L1, anti-programmed cell death protein-1 ligand; DDRgm, DNA damage repair gene mutations; PSMA, prostate-specific membrane antigen; CAR-T cells, chimeric antigen receptor-T cells; IL-2, Interleukin-2.

**Table 3:** Ongoing clinical studies with novel molecules/combinations.

Pathways Involved	Description	(NCT) No.	Phase	N
Anti-PD-1 Pembrolizumab + PARPi Olaparib	mCRPC, prior TXT and 1 ARSi therapy	02861573	1b/2	41
Anti-PD-L1 Avelumab + PARPi Talazoparib	*BRCA1/2* or *ATM* mutated (also mCRPC)	03565991	2	200
AZD4635 + anti-PD-L1 Durvalumab	Advanced solid tumors (also mCRPC)	02740985	1	307
Ciforadenant + anti-PD-L1 Atezolizumab	mCRPC and mRCC	02655822	1/1b	336
Anti-PD-L1 Avelumab + NKTR-214 (Arm A) + PARPi Talazoparib (Arm B) or ARSi Enzalutamide (Arm C)	Arm A: SCCHN; Arms B and C: mCRPC; Arm B enrolls DDR deficiency pts	04052204	1b/2	127
Anti-PD-L1 Avelumab-based combinations	Advanced malignancies (also mPCa)	03217747	1/2	184
Anti-PD-L1 Durvalumab + anti-CTLA-4 Tremelimumab + oral metronomic Vinorelbine	Solid tumors (also mPCa)	03518606	1/2	150
PSMA-CAR-T cells	mCRPC	04053062	1	12
CAR-T-PSMA-TGFβRDN cells	mCRPC	03089203	1	18
Iodine I-131-1095-radioconjugate anti-PSMA + Enzalutamide	mCRPC	03939689	2	120

Abbreviations: N, size; PARPi, poly (adenosine diphosphate [ADP]-ribose) polymerase inhibitor; ARSi, androgen receptor signaling inhibitor; mCRPC, metastatic castration-resistant prostate cancer; HR DDRgm, homologous recombination DNA damage repair gene mutation; anti-PD-1, anti-programmed cell death protein-1; pts, patients; TXT, docetaxel; anti-PD-L1, anti- programmed cell death protein-1 ligand; AZD4635, adenosine A2A receptor antagonist; Ciforadenant, anti-adenosine A2A receptor; mRCC, metastatic renal-cell carcinoma; NKTR-241, CD122-biased IL-2 receptor agonist; SCCHN, head and neck squamous cell carcinoma; DDR, DNA damage repair; mPCa, metastatic prostate cancer; anti-CTLA-4, anti-cytotoxic T lymphocyte antigen-4; PSMA-CAR-T, prostate-specific membrane antigen chimeric antigen receptor-T; mCRPC, metastatic castration-resistant prostate cancer; TGFβRDN, transforming growth factor-beta receptor dominant negative.

## Abbreviations

PCa	prostate cancer
AR	androgen receptor
ARS	AR-signaling
NGS	next-generation sequencing
DDR	DNA damage repair
mCRPC	metastatic castration-resistant PCa
CSS	cause-specific survival
PARP	poly (adenosine diphosphate [ADP]-ribose) polymerase
PD	programmed death
PSA	prostate-specific antigen
ICI	immune checkpoint inhibitors
SSB	single-stranded breaks
DSB	double-stranded breaks
HR	homologous recombination
NHEJ	non-homologous end joining
MMR	mismatch repair
PSMA	prostate-specific membrane antigen
CAR-T	chimeric antigen receptor-T
